# Peptides Derived from *Mycobacterium leprae* ML1601c Discriminate between Leprosy Patients and Healthy Endemic Controls

**DOI:** 10.1155/2012/132049

**Published:** 2012-01-29

**Authors:** Kidist Bobosha, Jolien J. van der Ploeg-van Schip, Danuza A. Esquenazi, Marjorie M. Guimarães, Marcia V. Martins, Yonas Bekele, Yonas Fantahun, Abraham Aseffa, Kees L. M. C. Franken, Ronaldo C. Gismondi, Maria C. V. Pessolani, Tom H. M. Ottenhoff, Geraldo M. B. Pereira, Annemieke Geluk

**Affiliations:** ^1^Department of Infectious Diseases, LUMC, P.O. Box 9600, 2300 RC Leiden, The Netherlands; ^2^Leprosy section, Armauer Hansen Research Institute, P.O. Box 1005, Addis Ababa, Ethiopia; ^3^The Laboratory of Cellular Microbiology, Oswaldo Cruz Institute, FIOCRUZ, 21040-360 Rio de Janeiro, RJ, Brazil; ^4^School of Medical Sciences, State University of Rio de Janeiro, 20550-170 Rio de Janeiro, RJ, Brazil

## Abstract

The stable incidence of new leprosy cases suggests that transmission of infection continues despite worldwide implementation of MDT. Thus, specific tools are needed to diagnose early stage *Mycobacterium leprae* infection, the likely sources of transmission. *M. leprae* antigens that induce T-cell responses in *M. leprae* exposed and/or infected individuals thus are major targets for new diagnostic tools. Previously, we showed that ML1601c was immunogenic in patients and healthy household contacts (HHC). However, some endemic controls (EC) also recognized this protein. To improve the diagnostic potential, IFN-**γ** responses to ML1601c peptides were assessed using PBMC from Brazilian leprosy patients and EC. Five ML1601c peptides only induced IFN-**γ** in patients and HHC. Moreover, 24-hour whole-blood assay (WBA), two ML1601c peptides could assess the level of *M. leprae* exposure in Ethiopian EC. Beside IFN-**γ**, also IP-10, IL-6, IL-1**β**, TNF-**α**, and MCP-1 were increased in EC from areas with high leprosy prevalence in response to these ML1601c peptides. Thus, ML1601c peptides may be useful for differentiating *M. leprae* exposed or infected individuals and can also be used to indicate the magnitude of *M. leprae* transmission even in the context of various HLA alleles as present in these different genetic backgrounds.

## 1. Introduction

Leprosy is a treatable infection caused by *Mycobacterium leprae* (*M. leprae*) involving skin and peripheral nerves and is influenced by genetic and environmental factors [1–3]. The infection can result in skin lesions, nerve degeneration, and deformities. Despite a spectacular decrease in global prevalence since 1982, transmission of leprosy is sustained as evidenced by the hundreds of thousand new cases of leprosy that keep being detected globally every year: 244,796 new cases of leprosy were detected during 2009 amongst whom 22,485 were children and the registered prevalence at the beginning of 2010 was 211,903 cases [4].

In Brazil, for example, the number of new cases detected during 2009 was 37,610 resulting in a registered prevalence of 38,179 at the end of first quarter of 2010 [4]. These figures demonstrate that *M. leprae*-infected contacts and persons with subclinical, undiagnosed leprosy, likely the major sources of unidentified transmission, are an incessant source of active transmission. Despite many efforts, prediction of disease development in affected individuals is still not possible nor can we detect asymptomatic *M. leprae* infection. Diagnosis of leprosy is usually based on clinical features and skin smear results including the number of skin lesions. *M. leprae* is not cultivable, and bacterial enumeration by microscopic examination is required for leprosy classification, choice in choosing and monitoring chemotherapy regimens, and diagnosis of relapse. However, detection and quantification using standard microscopy yields data of limited specificity and sensitivity. Thus, in order to complement current clinical methods, especially for PB patients, and to allow informed decision making on who needs treatment at a preclinical stage, several groups are investigating design of improved diagnostic tools. These tools will reduce transmission, prevent functional disabilities and stigmatizing deformities, and facilitate leprosy eradication, especially in individuals at risk for developing leprosy such as close contacts of leprosy patients.

Assays have been developed that detect *M. leprae*-specific IgM antibodies against PGL-I [5, 6], which are able to identify multibacillary (MB) leprosy patients (with strong humoral immunity to *M. leprae*), but these fail to detect most paucibacillary (PB) leprosy patients and leprosy patients' contacts as these typically develop strong cellular but not humoral immunity. One of the hurdles hampering T-cell-based diagnostic tests is that *M. leprae* antigens can cross-react at the T-cell level with antigens present in other mycobacteria, like *M. tuberculosis* or BCG even if the homology is relatively low as is the case for ESAT-6 and CFP-10 [7, 8]. Using comparative genomics, we previously identified candidate proteins highly restricted to *M. leprae* which showed promising features with respect to application in leprosy diagnostics [9, 10].

For specific detection of *M. tuberculosis* infection, commercially available IFN-*γ* release assays (IGRAs) like QuantiFERON-TB Gold have been developed [11]: these tests are based on cellular immune responses induced by a cocktail of peptides derived from ESAT-6 (Rv3875), CFP-10 (Rv3874), and TB7.7 (Rv2654) that are selectively expressed by *M. tuberculosis* and deleted from all (nonvirulent) BCG strains and most other NTMs [11]. This has inspired research into the feasibility of developing similar peptide-based assays for the identification of asymptomatic leprosy: encouraging results have been generated indicating that some synthetic peptides induce specific responses in individuals exposed to *M. leprae* and could potentially be developed into a rapid test for the detection of *M. leprae* infection [10, 12, 13]. In contrast to TB, however, ESAT-6- or CFP-10-derived peptides will not be useful due to the cross-reactive T-cell responses they induce in TB patients [7, 8].

Since T-cell reactivity to peptides are HLA-restricted [14–16], the use of a pool composed of several different *M. leprae* peptides, in analogy to the pool of peptides applied in the QuantiFERON-TB Gold tests, will increase sensitivity [17, 18], while avoiding T-cell cross-reactivity.

In order to improve sensitivity of a specific diagnostic peptide mixture, we have in this study extended the number of peptides with potential to distinguish exposure to *M. leprae* from BCG vaccination and exposure to other mycobacteria in a future diagnostic tool.

The protein ML1601c was previously identified by us as highly immunogenic in *M. leprae*-exposed Brazilian individuals [9], and although it does not contain a homologous sequence in *M. tuberculosis*, it does have an orthologue in *M. avium paratuberculosis*, MAP3249, which is 33% identical to ML1601c. To identify single peptides that are only recognized by *M. leprae*-exposed and/or infected individuals, we here analyzed IFN-*γ* production in Brazilian leprosy patients and controls in response to overlapping ML1601c peptides covering the whole protein.

## 2. Materials and Methods

### 2.1. Synthetic Peptides

ML1601c overlapping peptides (Table 1: two 19-mers with 9 amino acid overlap; eight 20-mers with 10 amino acid overlap; one 21-mer with 10 amino acids overlap) were purchased from Peptide 2.0 Inc. (Chantilly, VA, USA). Homogeneity and purity were confirmed by analytical HPLC and by mass spectrometry. Purity of all peptides was ≥80%. All impurities consist of shorter versions of the peptides caused by <100% coupling efficiency in each round of synthesis. Aliquots of identical batches of the synthetic peptides were tested in Brazil, Ethiopia, and The Netherlands.

### 2.2. Recombinant ML1601c Protein

The ML1601c gene was amplified by PCR from genomic DNA of *M. leprae* and cloned using the Gateway technology platform (Invitrogen, Carlsbad, CA, USA) with pDEST17 expression vector containing an N-terminal histidine tag (Invitrogen) [19]. Sequencing was performed on selected clones to confirm identity of all cloned DNA fragments. Recombinant proteins were overexpressed in *E. coli* BL21 (DE3) and purified as described to remove any traces of endotoxin. Each purified recombinant protein was analyzed by 12% SDS-PAGE followed by Coomassie Brilliant Blue staining and western blotting with an anti-His antibody (Invitrogen) to confirm size and purity. Endotoxin contents were below 50 IU per mg recombinant protein as tested using a Limulus Amebocyte Lysate (LAL) assay (Cambrex, East Rutherford, NJ, USA). Recombinant ML1601c protein was tested to exclude protein nonspecific T-cell stimulation and cellular toxicity in IFN-*γ* release assays using PBMC of *in vitro* PPD-negative, healthy Dutch donors recruited at the Sanquin Blood Bank, Leiden, The Netherlands. None of these controls had experienced any known prior contact with leprosy or TB patients.

### 2.3. *M. leprae* Whole-Cell Sonicate

Irradiated armadillo-derived *M. leprae* whole cells were probe-sonicated with a Sanyo sonicator to >95% breakage. This material was provided through the NIH/NIAID “Leprosy Research Support” Contract N01 AI-25469 from the Colorado State University (these reagents are now available through the Biodefense and Emerging Infections Research Resources Repository listed at http://www.beiresources.org/TBVTRMResearchMaterials/tabid/1431/Default.aspx).

### 2.4. Study Subjects

Twenty two Brazilian leprosy patients (11 paucibacillary (PB) leprosy patients and 11 multibacillary (MB)) were recruited from the Leprosy Out-Patient Unit, Leprosy Laboratory (Oswaldo Cruz Institute, Rio de Janeiro RJ, Brazil) and from the Duque de Caxias Outpatient Units (Health Department, Duque de Caxias, RJ, Brazil). Leprosy patients were diagnosed and classified based on clinical, bacteriological, and if possible histopathological findings. MB patients were treated with rifampicin, dapsone, and clofazimine. PB patients were treated with rifampicin and dapsone. All MB patients were skin-slit smear positive whereas PB patients were all skin-slit smear negative. All patients were tested before MDT was initiated. As controls, 19 healthy household contacts of MB leprosy patients (HHC), 8 tuberculosis patients (TB), and 17 healthy endemic controls (EC) were recruited from Duque de Caxias (*n* = 7) and the city of Rio de Janeiro (*n* = 10). Leprosy detection rates at the time of recruitment were 1.26 per 10,000 in Rio de Janeiro and 3.40 per 10,000 in Duque de Caxias (Ministry of Health of Brazil; http://www.datasus.gov.br/). From Ethiopia 34 healthy controls were tested: 18 EC_high_ were derived from a subcity of Addis Ababa (Kolfe Keranio Clinic) with a prevalence rate of 1.5 per 10,000 (72 in 465,811), whereas 16 EC_low_ were derived from areas with a prevalence rate of 0.36 per 10,000 (10 in 273,310). Prevalence rates in Ethiopia were calculated based on the number of patients in the health centers provided by the personnel of each health center. TB patients were recruited from the Ambulatory Service, District Hospital of Raphael de Paula e Souza, Rio de Janeiro RJ, Brazil. As nonendemic controls 21 Dutch healthy individuals (NEC) were recruited at the Sanquin Blood Bank, Leiden, The Netherlands. None of the NEC had experienced any known prior contact with leprosy patients. Informed consent was obtained from all individuals before venepuncture. Ethical approval of the study protocol was obtained through the appropriate local ethics committees.

### 2.5. Lymphocyte Stimulation Tests (LSTs)

Venous blood was obtained from study participants in heparinized tubes and PBMC isolated by Ficoll density centrifugation. PBMCs (1.5 × 10^6^ cells/mL) were plated in triplicate cultures in 96-well round-bottom plates (Costar Corporation, Cambridge, MA, USA) in 200 *μ*L/well of adoptive immunotherapy medium (AIM-V, Invitrogen, Carlsbad, CA, USA). Synthetic peptides, recombinant protein, *M. leprae* whole cell sonicate or PPD (purified protein derivative of *M. tuberculosis*, Mycos, Loveland, CO, USA) were added at final concentrations of 10 *μ*g/mL. As positive control stimuli SEB (staphylococcal enterotoxin B: 1 *μ*g/mL; Toxin Technology, Inc., Sarasota, FL, USA) or PHA (phytohemagglutinin; 2 *μ*g/mL; Sigma, St. Louis, MO, USA) was used. After 6 days of culture at 37°C at 5% CO_2_ and 90% relative humidity, 110 *μ*L supernatants were removed from each well; triplicates were pooled and frozen in aliquots at −20°C until further analysis.

### 2.6. Whole Blood Assays (WBAs)

Venous undiluted heparinized blood (450 *μ*L per well) was incubated in 48-well plates at 37°C at 5% CO_2_ and 70% relative humidity with 50 *μ*L of ML1601c peptide (p11 and p16) solution (10 *μ*g/mL final concentration). Blood was added to each well within 2 hours of collection. After 24 h of culture, 180 *μ*L of supernatants were removed from each well and frozen in aliquots at −20°C until further analysis.

### 2.7. IFN-*γ* ELISA

Detection of IFN-*γ* in culture supernatants of *in vitro* cultured cells was performed by ELISA (BD Bioscience) according to the manufacturer's instructions. OD values were converted into concentrations using Microplate Manager software, version 5.2.1 (Bio-Rad Laboratories, Veenendaal, The Netherlands). The cut-off value to define positive responses was set beforehand at 100 pg/mL. The assay sensitivity level was 20 pg/mL. Values for unstimulated whole blood cultures were typically <30 pg/mL.

### 2.8. Multiplex Determination of Cytokines and Chemokines

According to the manufacturer's guidelines, 18 inflammatory and immunomodulatory cytokines or chemokines (IL-1*β*, IL-2, IL-4, IL-5, IL-6, IL-7, IL-8, IL-10, IL-12p70, IL-13, IL-17, G-CSF, GM-CSF, IFN-*γ*, IP-10 (CXCL10), MCP-1 (CCL2), MIP-1*β* (CCL4), and TNF) were measured in unstimulated, antigen-stimulated, or mitogen-stimulated samples by Bio-Plex Suspension Array System powered by Luminex xMAP multiplex technology (Bio-Rad Laboratories, Veenendaal, The Netherlands) and analyzed with the Bio-Plex Manager Software 4.0 (Bio-Rad Laboratories, Veenendaal, The Netherlands). After prewetting the filter with assay solution, the beads were washed twice with washing solution using 96-well multiscreen filter plates (Millipore), an Aurum vacuum manifold and a vacuum pump (Bio-Rad Laboratories, Veenendaal, The Netherlands). Supernatant samples (50 *μ*L) were added to the plates, and the plates were incubated 45 minutes at room temperature in the dark at 300 rpm on a plate shaker. After three washes, 12.5 *μ*L detection antibody cocktail was added per well, and plates were incubated at room temperature in the dark for 30 minutes on a plate shaker. After three washes, 25 *μ*L streptavidin-PE solution was added per well and incubated for 10 minutes. After three washes, 100 *μ*L of assay buffer was added to each well and the plates were placed in the Bio-Plex System. From each well, a minimum of 100 analyte-specific beads were analyzed for fluorescence. A curve fit was applied to each standard curve according to the manufacturer's manual. Sample concentrations were interpolated from these standard curves. Analyte concentrations outside the upper or lower limits of quantification were assigned the values of the limits of quantification of the cytokine or chemokine.

### 2.9. Statistical Analysis

Differences in cytokine levels between groups were analyzed with the two-tailed Mann-Whitney *U* test for nonparametric distribution using Graph Pad Prism (version 5). *P* values were corrected for multiple comparisons. The statistical significance level used was *P* < 0.05.

### 2.10. Factor Analysis

The factor analysis technique was applied to evaluate the IFN-*γ* production levels induced by ML1601c and the ML1601c-derived peptides in order to identify the different patterns of response associated with these stimuli and to group together peptides inducing similar patterns of IFN-*γ* production. Three independent factors representing combinations of the original variables were determined. The factor loadings are the correlation coefficients between the original variables or IFN-*γ* responses to a given peptide and the factors (StatSoft, Inc. 2010, STATISTICA, data analysis software system, version 9; http://www.statsoft.com/).

## 3. Results

### 3.1. Identification of *M. leprae*-Specific T-Cell Epitopes of ML1601c in Brazilian Population Highly Endemic for Leprosy

**Figure 1:**
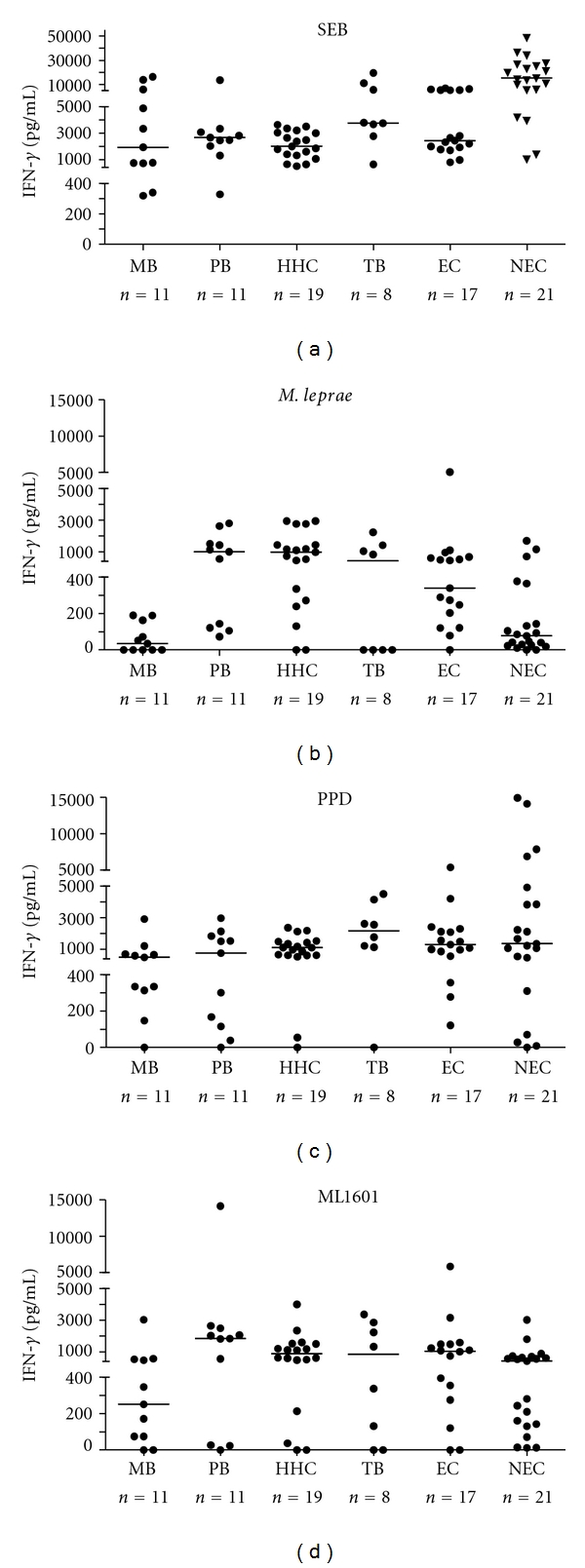
IFN-*γ* production by PBMC induced by SEB (a), PPD (b), *M. leprae *(c), and ML1601c recombinant protein (d) in MB (*n* = 11), PB (*n* = 11), HHC (*n* = 19), TB (*n* = 8), and EC (*n* = 17) from Brazil as well as in Dutch NEC (*n* = 21). For NEC, PHA was used instead of SEB. Values were corrected for background values. All background values were typically <20 pg/mL. Horizontal bars indicate median responses.

In view of its high immunogenicity in *M. leprae*-exposed individuals [9], the recombinant protein ML1601c was tested for induction of IFN-*γ* in PBMC derived from multibacillary (MB) and paucibacillary (PB) leprosy patients, healthy house contacts (HHCs), tuberculosis (TB) patients, and healthy controls (EC) from Brazil and from 21 Dutch (nonendemic) control (NEC) individuals (Figure 1). As controls, stimulation by *M. leprae* whole cell sonicate, purified protein derivative (PPD) of *M. tuberculosis* and staphylococcal enterotoxin B (SEB) was also analyzed. As can be seen from Figure 1 all groups responded well to the SEB control with median production >2400 pg/mL. For the Dutch NEC the positive control PHA was used instead of SEB, inducing overall higher IFN-*γ* responses in this group (Figure 1(a)). PPD was highly immunogenic in all groups as well, be it that IFN-*γ* responses to *M. leprae *showed more variability as only three individuals responded well in the NEC group, two intermediately, and 16 were nonresponders for *M. leprae*. Additionally, the MB patients did not or only barely respond to *M. leprae*, which is a general phenomenon for this type of leprosy patients. Similar to PPD, ML1601c protein did not induce significantly different IFN-*γ* production in EC compared to NEC nor compared to the HHC group, whereas MB patients again responded less well than the five other test groups. In summary, these data indicate that IFN-*γ* responses induced by ML1601c protein cannot be used to discriminate between *M. leprae*-exposed and nonexposed individuals.

**Figure 2:**
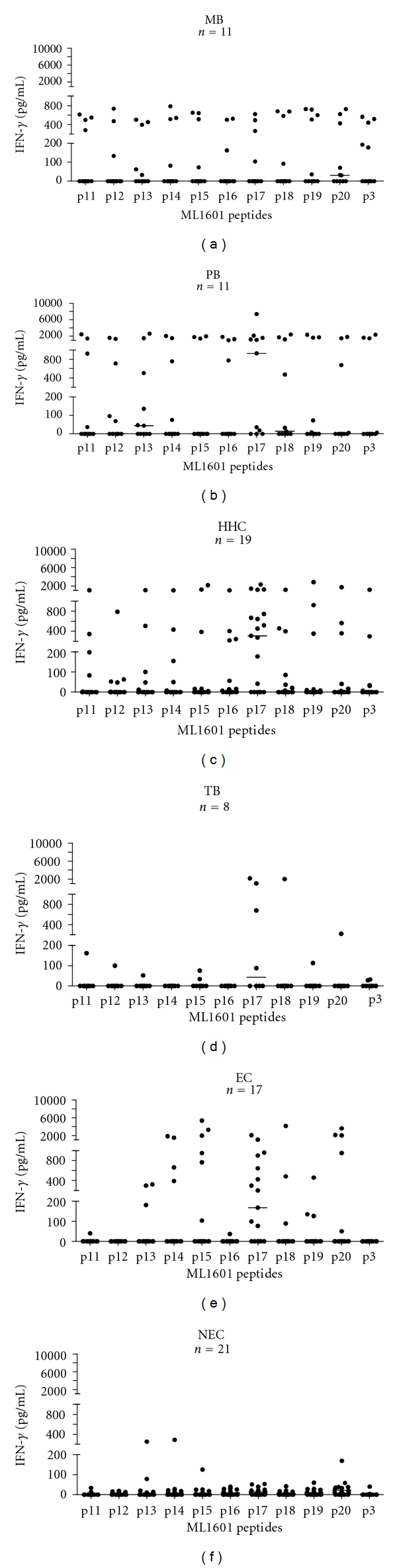
IFN-*γ* production by PBMC induced by ML1601c peptides (see Table 1) in Brazilian MB (*n* = 11), PB (*n* = 11), HHC (*n* = 19), TB (*n* = 8), and EC (*n* = 17) as well as Dutch NEC (*n* = 21). Values were corrected for background values. All background values were typically <20 pg/mL. Horizontal bars indicate median responses.

In view of this nondiscriminatory nature of the IFN-*γ* responses induced by the ML1601c protein in Brazilian individuals and due to the fact that ML1601c contains sequences similar or identical to *M. avium paratuberculosis* (MAP3249), peptides overlapping the entire sequence of ML1601c (Table 1) were synthesized. Analysis of IFN-*γ* responses induced by these ML1601c synthetic peptides in PBMC is shown in Figure 2: again, similar to stimulation with ML1601c protein, MB patients responded less well than PB patients and HHCs. In these latter two groups each ML1601c peptide was recognized by ≥1 (HHC) or ≥3 (PB) individuals with a maximum of 11 HHC recognizing p17. In contrast to the responses to the whole ML1601c protein, the synthetic peptides induced lower IFN-*γ* responses especially in NEC and TB, as none of the peptides were recognized by NEC and only p17 induced significant responses in three TB patients. Importantly, for the Brazilian EC only some peptides (p15, p17, p20, and to a lesser extent p18) induced significant responses in multiple donors. Thus, these data clearly indicate that peptide-induced IFN-*γ* production in response to ML1601c are more specific for and correspond with *M. leprae* exposure and/or infection.

### 3.2. Identification of ML1601c Peptides with Discriminatory Capacity

Since peptide responses are HLA restricted, a combination of multiple *M. leprae* peptides will be required to render a diagnostic test for leprosy broadly applicable. Thus, for the selection of peptides with the best performance in discriminating individuals with *M. leprae* infection and/or exposure based on their capacity to induce IFN-*γ* production in PBMC, a factor analysis was performed using the IFN-*γ* data (Figure 2) induced by all ML1601c peptides and the ML1601c protein (Table 2).

**Figure 3:**
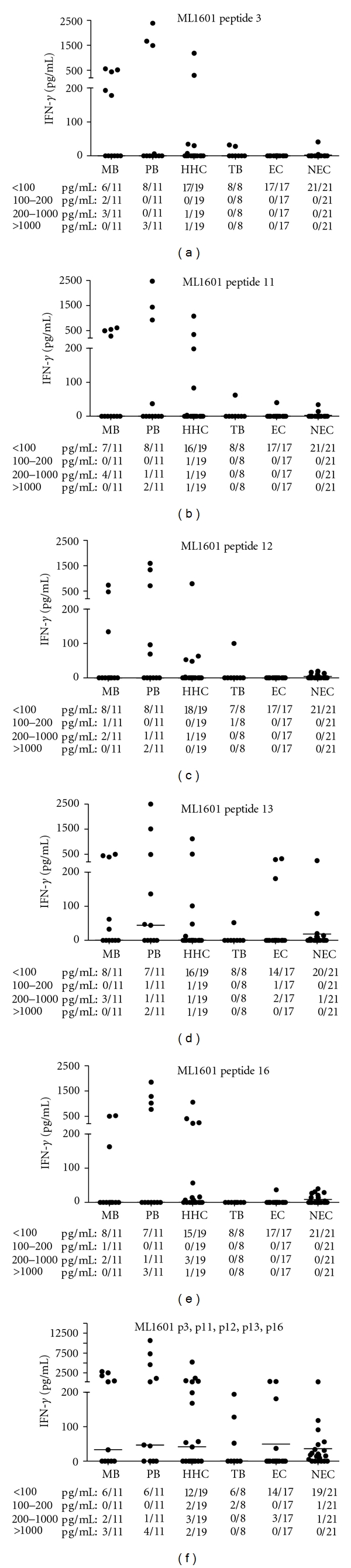
IFN-*γ* production by PBMC induced in all test groups by ML1601c peptides p3, p11, p12, p13, and p16 and the sum of the IFN-*γ* values for p3, p11, p12, p13, and p16 combined. The proportions of responders in each test group are indicated below the *x*-axis.

This type of analysis has the potential to group together peptides inducing comparable patterns of IFN-*γ* responses and as a consequence presenting high correlations (factor loadings) with the same factor. The 3 factors obtained from the IFN-*γ* responses to the ML1601c protein and the ML1601c peptides can in fact be linked to features relevant in the selection of peptides for use in diagnostic tests. Peptides presenting high correlations with factor 1 (factor loading > 0.8; p3, p11, p12, p13, and p16) induce high-level responses only in a subset of the exposed and/or infected individuals (MB, PB, HHC) but not on those for which exposure is less likely (EC), absent (NEC), or TB patients. Therefore, IFN-*γ* production induced by these peptides was depicted for each peptide separately as well as for the sum of the IFN-*γ* values for all five of these ML1601c peptides combined (Figure 3). This figure shows that IFN-*γ* levels in response to p13 were most frequent but were also observed in three EC and in one NEC, whereas p3, p11, and p16 showed very specific responses only in leprosy patients and in *M. leprae*-exposed HHC. This analysis clearly shows that *M. leprae*-specific IFN-*γ* responses can be induced selectively in PBMC derived from *M. leprae* exposed and/or infected individuals by peptides derived from a protein that is not uniquely present in the *M. leprae* genome. The ML1601c protein and p17 were highly correlated with factor 2. Responsiveness to these two stimuli was present in the exposed and/or infected groups, in the EC and in TB patients. So, the ML1601c protein and p17 (correlated to factor 2) are not useful antigens in terms of potentially discriminating *M. leprae* infection or disease. ML1601c p15 and p20 (correlated to factor 3) share with the factor 1 subset specificity for exposed and/or infected individuals. However, p15 and p20 also stimulate EC rendering these peptides not useful for leprosy diagnostics either.

### 3.3. Whole Blood Assays Using ML1601c Peptides in Ethiopian Healthy Controls

**Figure 4:**
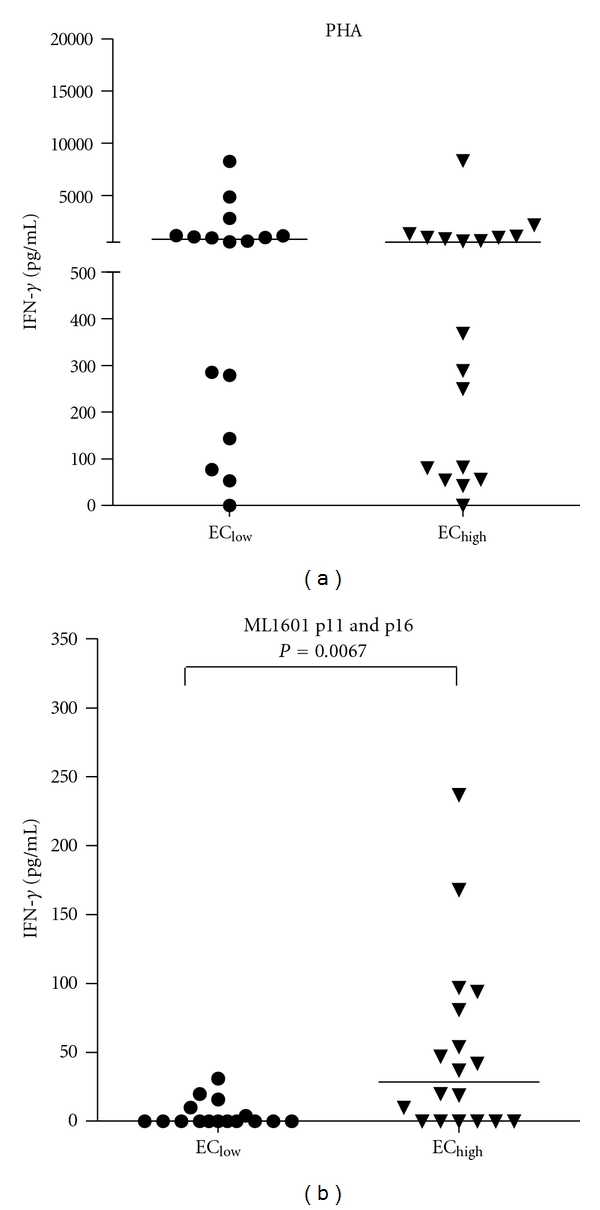
IFN-*γ* production in response to the positive control PHA (a) or a mix of ML1601c peptides no. 11 and no. 16 (b) measured after 24 h culture of undiluted whole blood derived from 34 Ethiopian healthy controls (ECs) derived from areas with low endemicity for leprosy (EC_low_:  *n* = 16) or from areas highly endemic for leprosy (EC_high_:  *n* = 18).

ML1601c p11 and p16 induced significant IFN-*γ* responses (>200 pg/mL; Figure 3) in 10 and 11 *M. leprae*-exposed or infected Brazilians, respectively, indicating recognition of these peptides in the context of various HLA alleles. Since one of the aims of this study was to develop field-friendly test that is world-wide applicable, IFN-*γ* production in response to a mix of these peptides was analyzed in a 24-hour WBA [20] using 34 healthy controls from areas in Ethiopia with different leprosy prevalence (Figure 4; EC_low_:  *n* = 16; EC_high_:  *n* = 18). Although both groups responded equally well to the positive control stimulus PHA (Figure 4(a)), there was a significant difference (*P* = 0.0067) between IFN-*γ* responses induced by the ML1601c peptide mix in individuals from an area with low endemicity (EC_low_) compared to those from an area with high endemicity (EC_high_). Thus, WBA shows that IFN-*γ* levels, induced by ML1601c peptides selected on the basis of IFN-*γ* responses induced in *M. leprae*-exposed or infected individuals in Brazil, can be detected as well in Ethiopian individuals exposed to *M. leprae*. IFN-*γ* responses to these peptides in a field-friendly 24-hour WBA can therefore be used as indication of the magnitude of the *M. leprae* transmission level in a given population.

### 3.4. Sequence Homology of ML1601c Peptides

Since *M. avium paratuberculosis* contains a homologue of ML1601c (MAP3249), the sequence of ML1601c was aligned with that of MAP3249, and the amino acid identity was determined for ML1601 peptides (Table 1). This alignment showed that the percentage identity in general was not very high, with 42% and 35% identical to MAP3249 for p11 and p16, respectively. Immunogenicity of the peptides did not correlate with the percentage identity, as p17 and p12 both had high percentage identical sequence (52% and 45%, resp.), but only p17 was recognized by many individuals. Thus, although a homologue of ML1601c protein is present in *M. avium paratuberculosis*, exact sequence identity is relatively low and allows induction of specific T-cell responses in *M. leprae*-exposed individuals by ML1601c peptides.

### 3.5. Multiplex Determination of Cytokines and Chemokines in Response to ML1601c Peptides

Immunological correlates of protection in leprosy are still lacking: although antigen-specific IFN-*γ* production is often used as a biomarker for *M. leprae* infection [9], it is possible that additional cytokines might allow more specific or qualitatively different detection of immune responses against *M. leprae *peptides. In order to further characterize the cellular immune response directed against ML1601c peptides, 15 additional cytokines and chemokines were tested in multiplex assays on identical supernatants as those used for IFN-*γ* (described above, Figure 4) obtained from the 24-hour WBA stimulated with a mix of ML1601c p11 and p16 using 34 healthy Ethiopian individuals.

**Figure 5:**
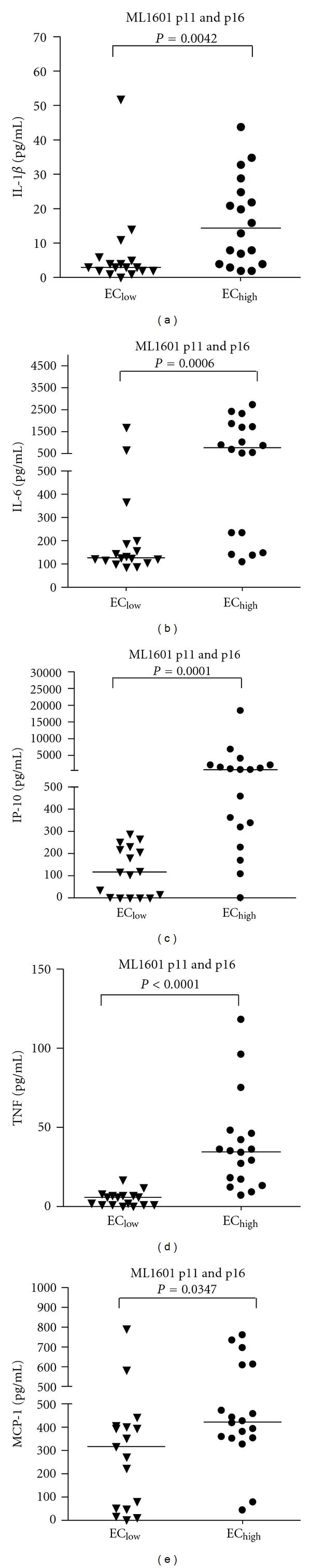
Production of IL-1*β* (a), IL-6 (b), IP-10 (c), TNF-*α* (d), and MCP-1 (e) in response to selected ML1601c peptides measured after 24 h culture of undiluted whole blood derived from 34 Ethiopian healthy controls (ECs) derived from areas with low endemicity for leprosy (EC_low_:  *n* = 16) or from areas highly endemic for leprosy (EC_high_:  *n* = 18).

Although hardly any responses were detected for IL-17, G-CSF, VEGF, IL-1*α*, IL-10, and GM-CSF nor any significant differences observed between EC_low_ and EC_high_ for the levels of IL-12, MIP-1*β*, MIG, and IL-8 (data not shown), significantly different levels were observed between these two groups when IL-1*β* (*P* = 0.0042), IL-6 (*P* = 0.0006), IP-10 (*P* = 0.0001), TNF-*α* (*P* < 0.0001), or MCP-1 (*P* = 0.0347) was measured (Figure 5). Thus, in addition to IFN-*γ*, detection of these cytokines can also be used to indicate the magnitude of the *M. leprae* transmission level in a given population. Whether or not such cytokine responses also indicate disease development or, alternatively, protection from disease will as yet have to be determined in longitudinal follow-up studies in HHC. Such studies are currently underway in highly leprosy endemic areas.

## 4. Discussion

It is quite clear that elimination of leprosy requires, in addition to multidrug therapy (MDT), novel diagnostic tools that allow early detection of preclinical *M. leprae* infection, likely the major source of unidentified transmission. Also, the fact that children are still developing leprosy suggests that MDT has not substantially reduced transmission [1, 2]. Therefore, identifying antigens that can be used as tools in diagnostic tests has been an important topic in leprosy research the last two decades.

In classical, PBMC-based IFN-*γ* release assays, *M. leprae* peptides have been shown to discriminate in a more specific fashion than proteins between *M. leprae*-exposed contacts and patients as opposed to healthy controls from the same endemic area [10, 12]. Our previous studies identified *M. leprae *peptides derived from proteins such as ML1989, ML1990, and ML2567 that induced IFN-*γ* in a 6-day proliferation assay using PBMC. The slight disadvantage of peptides though is that they usually induce significantly lower levels of IFN-*γ* than proteins, particularly when whole blood is used [13, 20]. This could, however, be inherent to the selected peptides as for TB diagnosis; the combination of >20 peptides is used successfully in WBA-based IGRA such as QuantiFERON-TB Gold assay. Therefore, more peptides, shared in different *M. leprae* strains that can be applied in diagnostic tools for leprosy, should be identified and tested in the context of various genetic backgrounds in South America, Asia, and Africa to enable development of a peptide-based WBA.

The Brazilian population can roughly be divided in three ethnic groups, namely, from Caucasian, indigenous, and African descent. Given this genetical diversity and the extraordinarily high leprosy endemicity compounded by poverty in several of its areas, Brazil is a suitable region for developing globally applicable T-cell-based diagnostic tools. Indeed, this study shows that even HLA-restricted,* M. leprae* peptides can be identified in a Brazilian population and applied to measure *M. leprae* exposure in an African population in Ethiopia. Two ML1601c peptides, p11 and p16, only induced IFN-*γ* production in PBMC from leprosy patients and HHC in Brazil and not, unlike ML1601c protein, in TB patients, EC, or NEC. The combination of these peptides could be applied in a field-friendly, 24 h WBA in Ethiopia to estimate exposure to *M. leprae*. This is consistent with the findings of other *M. leprae* peptides (Martins et al., submitted) thereby indicating that combinations of peptides can be designed and used efficiently to indicate substantial exposure to *M. leprae*.

The observation in this study that ML1601c protein induced significant IFN-*γ* responses, in EC, TB, as well as some NEC is in agreement with the finding that the use of recombinant proteins coincides with an increased risk of detecting cross-reactive T-cell responses irrespective of overall sequence homology. In addition, purification and quality control assays for recombinant proteins are more labor intensive than is the case for synthetic peptides. Therefore, despite the fact that T-cell responses to peptides are HLA restricted, which may limit the applicability of single peptides with respect to diagnostic T-cell-based assays in genetically diverse populations [21], a cocktail of *M. leprae* peptides can be used to identify *M. leprae* exposure in genetically different populations.

An alternative approach that we addressed here is that alternate cytokines or chemokines may be able to provide a distinction between progression to disease and containment of *M. leprae* infection. Therefore, we also tested supernatants of whole blood cultures stimulated with ML1601c p11 and p16 for 15 additional cytokines: significantly different levels were observed between EC_low_ and EC_high_ when IL-1*β* (*P* = 0.0042), IL-6 (*P* = 0.0006), IP-10 (*P* = 0.0001), TNF-*α* (*P* < 0.0001), or MCP-1 (*P* = 0.0347) was used as read-outs. Thus, in addition to IFN-*γ*, detection of these cytokines can also be used to estimate the magnitude of the *M. leprae* transmission level in a given population. The significant differences observed for both IL-1*β* and IL-6 suggest differences in the innate responses between the test groups [22]. For TB susceptibility it has been described that the polymorphism at the IL-1 locus influences the cytokine response and may be a determinant of delayed-type hypersensitivity and disease expression in human tuberculosis [23]. For leprosy, however, no association with IL-1*β* polymorphism has been described [24].

In combination with classical detection of anti-PGL-I IgM antibodies, *M. leprae* peptide-based WBA measuring cytokines will not only allow detection of most forms of leprosy (PB and MB) but may also identify those at risk of developing disease by detecting preclinical forms of leprosy, thereby enabling installment of MDT at an early stage. Additional *M. leprae *peptides will presumably be identified in the future, but, to ensure the success of developing an affordable, field-friendly test for the early diagnosis of leprosy, continued funding for these efforts will be critical.

## Figures and Tables

**Table 1:** ML1601c synthetic peptides.

Peptide no.	Amino acid sequence*	Amino acid identity**
11	A**HH**N**AHAA**PAF**L**WSGLVSA	42% (8/19)
12	F**L**W**S**GLVSA**AVL**IA**DGRGE**	52% (10/19)
13	**AVL**IA**DGRGE**DTYLPIISIY	40% (8/20)
14	DTYLPIISIYLA**R**GNE**L**KPN	10% (2/20)
15	LA**R**GNE**L**KPNPL**L**S**V**IYVE**H**	25% (5/20)
16	PL**L**S**V**IYVE**H**L**L**V**L**F**Y**Q**S**VG	35% (7/20)
17	L**L**V**L**F**Y**Q**S**VGD**H**C**GF**G**R**Y**D**F	45% (9/20)
18	D**H**C**GF**G**R**Y**D**FG**K**T**M**VL**A**C**YG**	50% (10/20)
19	G**K**T**M**VL**A**C**YG**CVGTRSL**L**SG	30% (6/20)
20	CVGTRSL**L**S**GR**DDDLVTSVP	15% (3/20)
3	RDDDLVTSVPPCGRASVVHRS	0% (0/21)

*Synthetic peptides overlapping ML1601c are shown in single-letter amino acid code.

**Amino acid sequences of ML1601c (*M. leprae *TN and BR4923) peptides were analyzed using BLAST (http://blast.ncbi.nlm.nih.gov/); amino acids that are identical to the MAP3249 are depicted in bold.

**Table 2:** Use of factor analysis for grouping together peptides and protein inducing similar patterns of IFN-*γ* responses*.

	Factor 1	Factor 2	Factor 3
p11	**0.9630**	0.0767	0.1875
p12	**0.9401**	0.0452	0.1643
p13	**0.9219**	0.0913	0.2498
p14	0.7010	0.0961	0.3563
p15	0.1877	−0.0058	**0.9473**
p16	**0.9243**	0.0214	0.1205
p17	0.1487	**0.9606**	0.0324
p18	0.5554	−0.0218	0.3223
p19	0.7157	−0.0095	0.3751
p20	0.3068	0.0327	**0.8787**
p3	**0.9524**	0.0701	0.1830
ML1601c	−0.0081	**0.9788**	−0.0103

*Values indicative of high correlation of a peptide IFN-*γ* response with one factor are depicted in bold. Factor analysis of the IFN-*γ* responses induced by the ML1601c protein and ML1601c peptides was performed with IFN-*γ* values observed in MB, PB, HHC, TB, and EC (STATISTICA, data analysis software system, version 9). The 3 factors are new and independent variables that capture the characteristics of the original variables (IFN-*γ* responses to the peptides and ML1601c protein in the different groups). The factor loadings indicative of the correlation of the IFN-*γ* responses induced by a peptide with each factor are shown. The 0.80000 value was arbitrarily selected for indicating a high correlation of one peptide with a given factor.
